# Chronic annular plaques with new peripheral follicular plugging

**DOI:** 10.1016/j.jdcr.2024.04.019

**Published:** 2024-05-10

**Authors:** Meridith S. Pensler, Alexandra C. Hristov, Maya S. Aravind

**Affiliations:** a University of Michigan Medical School, Ann Arbor, Michigan; b Department of Pathology, University of Michigan, Ann Arbor, Michigan; c Department of Dermatology, University of Michigan, Ann Arbor, Michigan

**Keywords:** annular, comedones, granuloma annulare, granulomatous, sun exposure

## Case presentation

**Fig 1:**
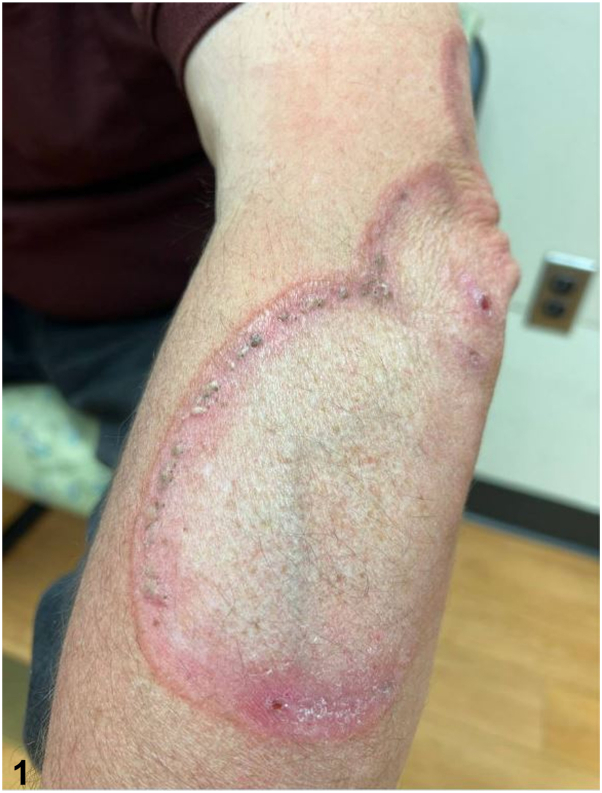

**Fig 2:**
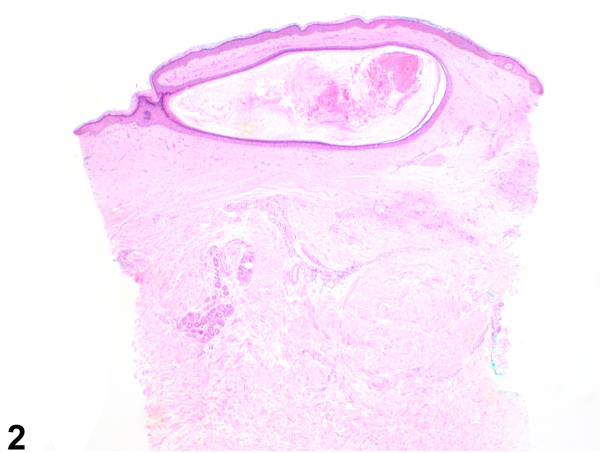

**Fig 3:**
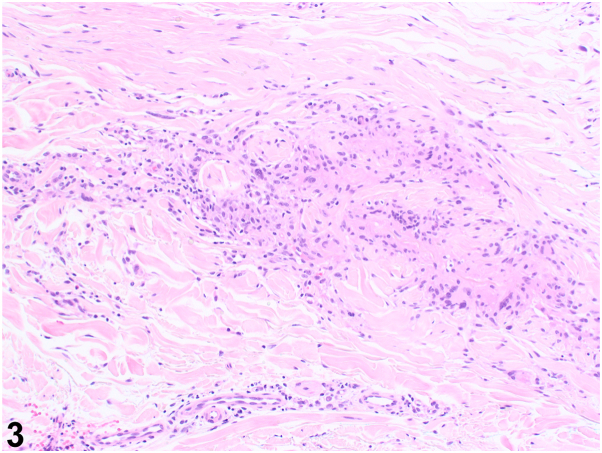

A 57-year-old White man presented with a 15-year history of bilateral hand and left forearm plaques. Six months prior to presentation to dermatology, he developed dark bumps at the border of the lesion on the left forearm (Fig 1) and right dorsal hand. He experienced intermittent pruritus. The patient was a former smoker with a past medical history notable for insulin-dependent type II diabetes. The patient worked as a truck driver for 28 years, with significant sun exposure. A punch biopsy was performed on a representative sample from his left forearm (Figs 2 and 3).

**Question 1: What is the most likely diagnosis?**

A. Elastosis perforans serpiginosa
B. Nevus comedonicus
C. Granuloma annulare (GA) with comedones
D. Cutaneous sarcoidosis
E. Tinea corporis

**Answers:**

A. Elastosis perforans serpiginosa – Incorrect. Elastosis perforans serpiginosa is a perforating disease with histology showing transepidermal elastin elimination, not palisading granulomas as seen in this patient.
B. Nevus comedonicus – Incorrect. This condition presents with grouped comedone-like keratinous plugs often in a linear, not annular, plaque. Also, it usually appears at birth or before age 10.
C. GA with comedones – Correct. This patient’s erythematous annular plaques had multiple open comedones at the peripheral border. Biopsy demonstrated palisaded granulomatous dermatitis with adjacent comedonal cyst and increased mucin, the latter highlighted by colloidal iron. In addition, CD123 marked the palisaded histiocytes.
D. Cutaneous sarcoidosis – Incorrect. Sarcoidosis is a granuloma forming skin condition. However, mucin would not be present on histopathology as seen in this patient.^1^
E. Tinea corporis – Incorrect. Tinea corporis is a fungal infection that presents with annular plaques. However, this patient’s skin lesions do not have peripheral scale that is typically associated with this infection.

**Question 2: What risk factor in this patient’s history is most likely associated with comedone formation?**

A. Type II diabetes
B. Sun exposure/occupation
C. Age
D. Sex
E. Race

**Answers:**

A. Type II diabetes – Incorrect. Although there has been debate in the literature about the association of type II diabetes with GA, it is not a specific risk factor for comedone formation.^2^
B. Sun exposure/occupation – Correct. Comedones are thought to develop due to UV-B–induced sebaceous gland hyperplasia and elastin degeneration. The elastin degeneration compounded with collagen degeneration histologically seen in GA allows the comedone to grow without resistance.^3^ This patient’s occupation involved driving with his left arm photo-exposed, resulting in the majority of comedones at this location.
C. Age – Incorrect. Although this patient’s older age correlates with increased sun exposure, his age does not directly explain the pathophysiology behind comedone formation.
D. Sex – Incorrect. GA has a predilection for females;^1^ however, sex in itself has not been shown to be correlated with comedone formation.
E. Race – Incorrect. GA is more common in White patients than Black, Hispanic, or Asian patients; however, there is no evidence to suggest that race is directly correlated with comedone formation.

**Question 3: Which of the following therapies have *not* shown to improve either localized or generalized GA?**

A. Intralesional or topical steroids
B. Hydroxychloroquine
C. Potassium iodide
D. Phototherapy
E. Apremilast

**Answers:**

A. Intralesional or topical steroids – Incorrect. Although patients with GA experience mixed responses, local corticosteroids are typically used as the first line treatment for localized GA.^1^
B. Hydroxychloroquine – Incorrect. Hydroxychloroquine is an antimalarial agent shown to be effective in treating generalized GA.^4^
C. Potassium iodide – Correct. Although there are some distant reports of potassium iodide being an effective treatment, a double-blind, placebo-controlled study did not find any advantage over the placebo in treating generalized GA.^5^
D. Phototherapy – Incorrect. There is evidence that phototherapy is effective at treating both localized and generalized GA.^4^ However, not all patients respond to this treatment and it is financially burdensome.^1^
E. Apremilast – Incorrect. Apremilast is a phosphodiesterase-4 that has shown to be beneficial in treating generalized GA in multiple case reports.^1^

## Conflicts of interest

None disclosed.
